# Using cost-effectiveness analysis to support decision making in resource poor settings: methods and practical challenges

**DOI:** 10.1186/1472-6963-14-S2-O15

**Published:** 2014-07-07

**Authors:** Mark Sculpher

**Affiliations:** 1 Centre for Health Economics, University of York, UK

## 

In the context of finite budgets, resource allocation in health care is a challenge to all health care systems. This is particularly pronounced in low income countries where opportunities to improve population health are even more limited by resource constraints. Cost-effectiveness analysis has been increasingly used to inform decisions about the choice of interventions and programmes in those settings. The application of the tools of CEA needs, however, to reflect a range of factors which can differ from those in more affluent jurisdictions. These include multiple sources of funding including those from donors, the existence of a number of constraints in addition to those relating to budgets, expectations of cost-effectiveness thresholds which have no empirical basis and marked uncertainty associated with locally-applicable evidence. There also exists a range of practical challenges in many countries, most notably the lack of capacity in skilled analysts. The Bill and Melinda Gates Foundation recently funded an initiative to standardize the methods of economic evaluation used in research projects that it funds. The presentation discusses the principles set out in that project, and considers the emerging agenda about methods research relating to economic evaluation in low income countries.

